# Preoperative hyponatremia predicts complications in older patients undergoing digestive tract surgery: a propensity score matching analysis

**DOI:** 10.1007/s41999-021-00559-4

**Published:** 2021-09-23

**Authors:** Chun-Qing Li, Chen Zhang, Fan Yu, Hao Kong, Chun-Mei Deng

**Affiliations:** Department of Anesthesiology and Critical Care Medicine, Peking University First Hospital, No. 8, Xishiku Street, Beijing, 100034 China

**Keywords:** Hyponatremia, Older patient, Digestive tract surgery, Postoperative complications, Propensity score

## Abstract

**Purpose:**

Whether preoperative hyponatremia increases the risk of postoperative complications in older patients undergoing digestive tract surgery remains unclear. The purpose of the study was to investigate the association between preoperative hyponatremia and life-threatening postoperative complications (including death) among older patients undergoing digestive tract surgery.

**Methods:**

This was a propensity score-matched, retrospective cohort study. We recruited older patients (≥ 65 years of age) who underwent digestive tract surgery in our institution. The propensity score matching (PSM; 1:2) was used to balance a range of covariates between patients with preoperative hyponatremia (serum sodium [Na^+^] levels < 135 mmol/L) and those with normal [Na^+^] levels (135–145 mmol/L). The primary outcome was the occurrence of life-threatening postoperative complications and mortality (i.e., Clavien–Dindo grade IV and V [CD IV and V] complications) during hospital stay. The relationship between preoperative hyponatremia and the development of CD IV and V complications was identified with logistic regression analysis.

**Results:**

A total of 1076 patients were analyzed (mean age 73.9 years; 421 female [39.1%]). Preoperative hyponatremia was identified in 122 patients. After matching, 312 patients were included in the analyses (104 patients with preoperative hyponatremia and 208 patients with normal [Na^+^] levels; mean age, 76.3 years; 130 female [41.7%]). When compared with normal [Na^+^] levels, preoperative hyponatremia was associated with an increased risk of CD IV and V complications (odds ratio [OR] 2.082, 95% confidential interval [CI] 1.041–4.164, *P* = 0.038). Preoperative hyponatremia was also significantly associated with a higher risk of postoperative infectious complications (OR 2.158, 95% CI 1.138–4.091, *P* = 0.018).

**Conclusion:**

Preoperative hyponatremia can predict an increased risk of life-threatening postoperative complications and mortality in older patients undergoing digestive tract surgery.

**Supplementary Information:**

The online version contains supplementary material available at 10.1007/s41999-021-00559-4.

## Key summary points

**Aim:**

This study aimed to investigate the association between preoperative hyponatremia and life-threatening postoperative complications (including death) among older patients undergoing digestive tract surgery.

**Findings:**

Preoperative hyponatremia was associated with an increased risk of life-threatening postoperative complications and mortality in older patients undergoing digestive tract surgery. Preoperative hyponatremia was also correlated with a higher risk of postoperative infectious complications.

**Message:**

Preoperative hyponatremia can predict the development of life-threatening postoperative complications and mortality in older patients undergoing digestive tract surgery.

**Supplementary Information:**

The online version contains supplementary material available at 10.1007/s41999-021-00559-4.

## Introduction

Hyponatremia, defined as a serum sodium ([Na^+^]) concentration < 135 mmol/L, is a common electrolyte imbalance that occurs in many clinical settings [1]. Hyponatremia is caused by a relative excess of water compared to sodium in the extracellular fluid (ECF), reflecting the pathophysiologic alternations in water homeostasis. Sodium and its accompanying anions are the major osmotically active plasma solutes and influence the osmotic movement of water across cell membranes; thus, true hyponatremia is most commonly associated with hypo-osmolality (i.e., hypotonic hyponatremia) [2, 3]. In clinical practice, two situations where hyponatremia is not accompanied by hypo-osmolality, i.e., pseudohyponatremia and translocational hyponatremia, should be considered in the differential diagnosis of true hyponatremia. Pseudohyponatremia, as a type of isotonic hyponatremia, is derived from a laboratory artifact: excess plasma protein or lipid reduces the water content of a given volume of plasma, thereby reducing the [Na^+^] concentration per unit of plasma; in fact, the [Na^+^] concentration in the water phase is normal (e.g., in hyperproteinemia or hypertriglyceridemia) [2–4]. Translocational hyponatremia, as a type of hypertonic or isotonic hyponatremia, occurs with the presence of large amounts of osmotically-active solutes, which can lead to fluid shift from intracellular to extracellular space, thus yielding dilutional hyponatremia (e.g., in hyperglycemia) [2–4]. Depending on the ECF volume status, the hypotonic hyponatremia can be divided into three categories: hypovolemic (decreased total body water [TBW] and ECF volume with a greater decrease in total body sodium, caused by gastrointestinal fluid losses, diuretic therapy, etc.), euvolemic (increased TBW and normal ECF volume with stable total body sodium, caused by the syndrome of inappropriate antidiuretic hormone [SIADH], glucocorticoid deficiency, etc.), and hypervolemic hyponatremia (increased total body sodium with a greater increase in TBW and ECF volume, caused by heart failure, cirrhosis, kidney diseases, etc.) [2, 3]. Increased arginine vasopressin (AVP) secretion is considered to be the most common mechanism of hyponatremia [2, 5]. Given that hyponatremia can be induced by various diseases or abnormal conditions, it is usually regarded as the surrogate marker for certain underlying diseases or pathophysiological status such as frailty [2, 6, 7].

The prevalence of hyponatremia varies dramatically and depends on different clinical settings, patient populations, and the precise definition of hyponatremia adopted [6]. The highest prevalence of hyponatremia in hospitalized patients is reported in older patients admitted to geriatric wards (22.2%) [1]. Older patients are more likely to develop hyponatremia than younger patients; this can be attributed to the accumulated comorbidity burden, polypharmacy, and age-related physiological changes (such as idiopathic SIADH, deficiency in water-excretory capacity, and frailty syndrome) [7–9]. Although hyponatremia was not an isolated disease, studies have suggested that hyponatremia is an independent predictor for increased mortality risk, even in mild or borderline cases [10, 11]. The adverse impacts of preoperative hyponatremia on postoperative outcomes have been investigated among diverse surgical populations, such as those undergoing orthopedic, cardiac, or head and neck surgery [12–16]. However, whether preoperative hyponatremia increases the risk of postoperative complications remains controversial [13, 16].

Among older inpatients, those scheduled for digestive tract surgery are, unsurprisingly, prone to developing hyponatremia. Besides the common predisposing factors in older individuals, the increased nonosmotic AVP secretion stimulated by volume depletion (due to gastrointestinal fluid losses or the third spacing of fluids) and the reduced oral sodium intake may contribute to the development of hyponatremia in this patient population [2]. So far, no study has explored the association between preoperative hyponatremia and adverse outcomes in older patients undergoing digestive tract surgery. We hypothesized that preoperative hyponatremia could predict an increased risk of life-threatening complications and mortality in this older surgical population. The primary objective of the present study was to explore the association between preoperative hyponatremia and the risk of life-threatening postoperative complications and mortality in older patients undergoing digestive tract surgery.

## Methods

### Study design

This was a propensity score-matched, retrospective cohort study that was carried out in a single center. The study protocol was approved by the Biomedical Research Ethics Committee of Peking University First Hospital (Reference: 2019 [296]; Beijing, China). Individual written informed consent was waived on account of the retrospective study design and that no patient follow-up was performed. All perioperative data were acquired from our electronic medical records system. All personal data were kept confidential.

### Patient selection

We included all older patients (≥ 65 years of age) who underwent digestive tract surgery in Beijing University First Hospital between 1st January 2017 and 31st December 2018. Exclusion criteria included the following: (1) absence of serum [Na^+^] measurements within three days before surgery; (2) a preoperative serum [Na^+^] level ≥ 146 mmol/L; (3) missing or incomplete perioperative data.

### Definition of preoperative hyponatremia

The primary independent variable of interest was preoperative hyponatremia. Preoperative hyponatremia was defined as a serum [Na^+^] concentration < 135 mmol/L reported in the most recent laboratory test within 3 days before surgery. Normal serum [Na^+^] level was defined as 135–145 mmol/L. Patients with preoperative serum [Na^+^] levels ≥ 146 mmol/L were excluded, because the investigation of high levels of [Na^+^] was outside the range of the present study. Based on baseline serum [Na^+^] levels, the patients included in this study were classified into two groups: a hyponatremia group and a normal [Na^+^] group.

### Covariates

Baseline and intraoperative data were collected, including (1) demographic characteristics (age, sex, and body mass index [BMI]); (2) other preoperative variables (frailty status [assessed by using 11-item modified frailty index, mFI] [17], American Society of Anesthesiologists [ASA] Classification, Charlson Comorbidity Index [CCI] score, New York Heart Association [NYHA] functional classification, functional ability to perform basic activities of daily living [basic ADLs, evaluated with the Barthel Index scale] [18], recent weight loss, preoperative blood transfusion, and major comorbidities); and (3) intraoperative variables (indication for surgery [malignancy or benign], type of surgery [gastric, intestinal, hepatopancreatobiliary, and simple general surgeries], emergency surgery or not, surgical approach [laparotomy or laparoscopy], anesthetic method, duration of surgery, and intraoperative blood transfusion). Simple general surgeries were defined as low-risk, less-damaging, and 23-h-stay digestive tract surgeries, such as laparoscopic cholecystectomy, hepatic cyst fenestration, hernia repair, and appendectomy.

### Postoperative outcomes

Postoperative complications were evaluated with the Clavien**–**Dindo (CD) classification system (Supplementary Table 1) [19]. The primary outcome was the occurrence of life-threatening complications requiring intermediate care/intensive-care unit [ICU] management and mortality (i.e., CD IV and V complications) during hospital stay. If more than one surgical procedure occurred during hospitalization, we only analyzed the first round of surgery. If multiple complications occurred in a particular patient, only the most severe complication was considered in our analysis. The diagnostic criteria for postoperative complications are shown in Supplementary Table 2.

Secondary outcomes included the occurrence of specific complications during the hospital stay, ICU admission after surgery, prolonged length of stay (LOS) in hospital, and adverse discharge disposition. The specific complications referred to those classified into CD grade II or greater rather than CD grade IV and V due to the low numbers of patients with specific CD IV and V complications. The specific complications included cardiovascular, respiratory, neurological, renal, hepatic, thromboembolic, infectious, and gastrointestinal complications. Of these, the infectious complications included abdominal abscess, superficial or deep incisional infection, respiratory infection, urinary tract infection, sepsis, and infectious diarrhea. Detailed definitions of specific complications occurring in this study cohort are shown in Supplementary Table 2. Prolonged LOS in hospital was defined as greater than the 75th percentiles of LOS for each type of surgery. Adverse discharge disposition was defined as discharge to destinations other than home (e.g., a long- or short-term rehabilitation facility). To eliminate the risk of diagnostic suspicion bias, independent variables and outcomes were collected by different investigators who were highly trained and blinded to the objective of the study.

### Statistical analysis

Hyponatremic patients were matched to those with normal [Na^+^] levels in a 1:2 ratio by applying the nearest neighbor matching method without replacement, with a caliper width equal to 0.2 of the standard deviation of the logit of the propensity score. The propensity score matching (PSM) process was carried out using a logistic regression model with exposure assignment (preoperative hyponatremia or normal [Na^+^]) as a dependent variable and potential confounders as independent variables. The confounders included in the matching model were considered to be related to the primary and secondary outcomes of the study, including (1) demographic characteristics (age, sex, and BMI); (2) general health status (frailty status represented by 11-item mFI score, ASA classification, CCI score, NYHA classification, and presence of impaired ADLs); (3) major comorbidities and other baseline factors (hypertension, diabetes mellitus, coronary artery disease, arrhythmia, previous stroke, pulmonary diseases, hepatic insufficiency, renal dysfunction, preoperative infection, hypoalbuminemia, anemia, preoperative blood transfusion, and recent weight loss); and (4) intraoperative factors (malignancy or not, type of surgery, emergency surgery or not, surgical approach, anesthetic method, duration of surgery, and intraoperative blood transfusion). Absolute standardized differences (ASDs) for the covariates were calculated before and after matching, with less than 0.1 (10%) indicating a balance between the two groups [20].

Primary and secondary outcomes were compared between two groups before and after matching, using the *χ*^2^ test, continuity-corrected *χ*^2^ test, or Fisher’s exact test. In the matched cohort, logistic regression analysis was used to evaluate the odds ratios (ORs) of hyponatremia for predicting primary or secondary outcomes. We also performed a sensitivity analysis in the pre-matched cohort (*n* = 1076) to verify the robustness of our main results. In the entire cohort, covariates in association with the primary outcome were screened using univariate logistic regression analyses. After testing for multicollinearity, factors with *P* values < 0.05 in the univariate analyses were included in the multivariate logistic regression model to identify predictors of CD IV and V complications with the Wald (backward) method.

Two-tailed *P* values < 0.05 were considered to be statistically significant. All statistical analyses were performed with SPSS version 22 software (SPSS, Inc, Chicago, IL) and the free software package “R” version 2.15.3 including “Matchit” and “ROC” plugins.

## Results

### Patient recruitment

**Fig. 1:**
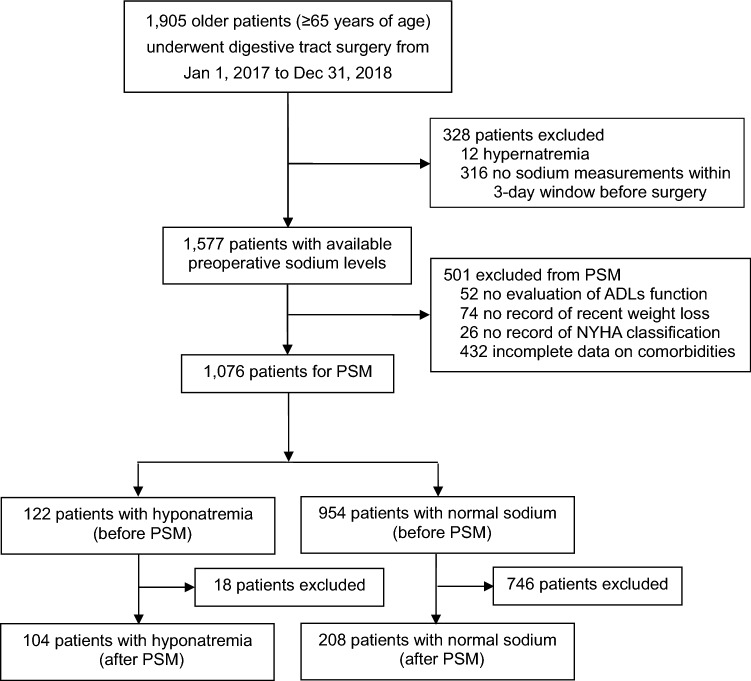
Flowchart of the study. PSM, propensity score matching; ADLs, activities of daily living; NYHA, New York Heart Association

We recruited a total of 1905 patients who were ≥ 65 years of age and underwent digestive tract surgery between 1st January 2017 and 31st December 2018. After excluding patients with hypernatremia and those lacking serum [Na^+^] levels within 3 days before surgery, we had a cohort of 1577 patients. Of these, 1076 patients had a complete dataset available for matching. Of the 1076 patients, 122 patients (11.3% [122/1076]) were found to have preoperative hyponatremia, and 954 patients (88.7% [954/1076]) had normal [Na^+^] levels. After matching, 104 patients remained in the hyponatremia group, and 208 patients remained in the normal [Na^+^] group, thus yielding a cohort of 312 patients for final analyses (Fig. 1).

### Patient characteristics before and after matching

Before matching, we found that the hyponatremic patients were older, and had worse general health status (higher ASA/NYHA classification and mFI score, and a greater rate of impaired ADLs), more comorbidities (higher rates of hypertension, arrhythmia, hepatic insufficiency, renal dysfunction, preoperative infection, hypoalbuminemia, and anemia), but lower BMI and a lower rate of malignant tumor than those with normal [Na^+^] levels (All ASDs ≥ 0.1). In addition, hyponatremic patients were more likely to experience hepatopancreatobiliary surgery, emergency surgery, laparotomy, general anesthesia, and preoperative or intraoperative blood transfusion, and received less gastric surgery (All ASDs ≥ 0.1; Supplementary Table 3). After matching, the two groups were adequately balanced (All ASDs < 0.1; Table 1).

**Table 1:** Baseline and intraoperative characteristics of matched cohort

Covariates	Hyponatremia (*n* = 104)	Normal sodium (*n* = 208)	ASD^a^
Preoperative factors			
Age, years	75.9 ± 6.5	76.4 ± 7.0	0.081
Female	42 (40.4%)	88 (42.3%)	0.039
Body mass index, kg/m^2^	22.4 ± 3.3	22.3 ± 3.5	0.038
Modified frailty index			
0.00	16 (15.4%)	28 (13.5%)	0.053
0.09	33 (31.7%)	69 (33.2%)	0.031
0.18	23 (22.1%)	49 (23.6%)	0.035
0.27	18 (17.3%)	35 (16.8%)	0.013
0.36	10 (9.6%)	17 (8.2%)	0.049
≥ 0.45	4 (3.8%)	10 (4.8%)	0.050
ASA classification			
I/II	48 (46.2%)	96 (46.2%)	0.000
III	51 (49.0%)	102 (49.0%)	0.000
IV/V	5 (4.8%)	10 (4.8%)	0.000
CCI score	2 (0, 4)	2 (0, 4)	0.087
NYHA classification			
I	35 (33.7%)	74 (35.6%)	0.041
II	62 (59.6%)	117 (56.3%)	0.068
III	7 (6.7%)	17 (8.2%)	0.057
Impaired ADLs^b^	43 (41.3%)	85 (40.9%)	0.010
Recent weight loss^c^	26 (25.0%)	47 (22.6%)	0.055
Hypertension	58 (55.8%)	112 (53.8%)	0.039
Diabetes mellitus	26 (25.0%)	53 (25.5%)	0.011
Coronary artery disease	17 (16.3%)	34 (16.3%)	0.000
Arrhythmia^d^	14 (13.5%)	29 (13.9%)	0.014
Previous stroke	20 (19.2%)	41 (19.7%)	0.012
Pulmonary diseases^e^	14 (13.5%)	30 (14.4%)	0.028
Hepatic insufficiency^f^	11 (10.6%)	17 (8.2%)	0.078
Renal dysfunction^g^	7 (6.7%)	14 (6.7%)	0.000
Preoperative infections^h^	13 (12.5%)	21 (10.1%)	0.072
Hypoalbuminemia^i^	69 (66.3%)	142 (68.3%)	0.041
Anemia^j^	43 (41.3%)	95 (45.7%)	0.087
Preoperative transfusion	11 (10.6%)	25 (12.0%)	0.047
Intraoperative factors			
Indication for surgery			
Malignancy	69 (66.3%)	130 (62.5%)	0.081
Benign	35 (33.7%)	78 (37.5%)	0.081
Type of surgery			
Simple general^k^	7 (6.7%)	16 (7.7%)	0.038
Gastric	12 (11.5%)	28 (13.5%)	0.060
Intestinal	66 (63.5%)	128 (61.5%)	0.040
Hepatopancreatobiliary	19 (18.3%)	36 (17.3%)	0.025
Emergency surgery	24 (23.1%)	41 (19.7%)	0.079
Surgical approach			
Laparotomy	29 (27.9%)	58 (27.9%)	0.000
Laparoscopy	75 (72.1%)	150 (72.1%)	0.000
Anesthetic method			
General aesthesia	72 (69.2%)	135 (64.9%)	0.093
Others^l^	32 (30.8%)	73 (35.1%)	0.093
Duration of surgery, min	169 (127, 240)	179 (130, 240)	0.011
Intraoperative transfusion	12 (11.5%)	24 (11.5%)	0.000

Data are presented as number (%), mean ± SD, or median (interquartile range)

*ASD* absolute standardized difference, *ASA* American Society of Anesthesiologists, *CCI* Charlson Comorbidity Index, *NYHA* New York Heart Association, *ADLs* activities of daily living

^a^An ASD < 0.1 was considered balanced [20]

^b^Requiring partial or total assistance from other people for basic ADLs (such as feeding, bathing, grooming, continence, transfers, etc.); evaluated with the Barthel Index scale [18]

^c^Unintentional body weight loss ≥ 10% of the baseline weight within 6 months, or ≥ 5% within 3 months, or ≥ 2% within 1 month

^d^Arrhythmia that required medical or interventional therapy

^e^Include chronic obstructive pulmonary disease and asthma

^f^Defined as Child–Pugh class B and C

^g^Defined as estimated glomerular filtration rate (eGFR) < 45 ml/min/1.73 m^2^ or on dialysis

^h^Include preoperative intra-abdominal infection and respiratory infection

^i^Defined as preoperative serum albumin level < 40 g/L

^j^Diagnosed according to the last laboratory test results before surgery: Male: < 120 g/L, female < 110 g/L

^k^Defined as low-risk, less-damaging, and 23-h-stay digestive tract surgeries, such as laparoscopic cholecystectomy, hepatic cyst fenestration, hernia repair, and appendectomy

^l^Include combined epidural-general anesthesia, combined peripheral nerve block-general anesthesia, and epidural/combined spinal-epidural anesthesia

### Primary outcomes after matching

After matching, hyponatremic patients had a significantly higher incidence of CD IV and V complications than those with normal [Na^+^] levels (17.3% vs. 9.1%, *P* = 0.035; Table 2). The logistic regression analysis showed that the hyponatremia group had a twofold higher risk of CD IV and V complications than the normal [Na^+^] group (OR 2.082, 95% CI 1.041–4.164, *P* = 0.038; Table 3).

**Table 2:** Postoperative outcomes of matched cohort

Outcomes	Hyponatremia (*n* = 104)	Normal sodium (*n* = 208)	*P* value
Primary outcome			
CD IV and V complications	18 (17.3%)	19 (9.1%)	**0.035**
CD IV complications	15 (14.4%)	14 (6.7%)	**0.027**
CD V complication	3 (2.9%)	5 (2.4%)	> 0.999
Secondary outcomes			
CD II or greater complications			
Cardiovascular	15 (14.4%)	26 (12.5%)	0.636
Respiratory	17 (16.3%)	21 (10.1%)	0.112
Neurological	5 (4.8%)	6 (2.9%)	0.587
Renal	5 (4.8%)	9 (4.3%)	> 0.999
Hepatic	1 (1.0%)	4 (1.9%)	0.873
Thromboembolic	2 (1.9%)	8 (3.8%)	0.570
Infectious	22 (21.2%)	23 (11.1%)	**0.017**
Gastrointestinal	14 (13.5%)	30 (14.4%)	0.818
ICU admission after surgery	40 (38.5%)	78 (37.5%)	0.869
Prolonged LOS in hospital	27 (26.0%))	57 (27.4%)	0.787
Adverse discharge disposition	7 (6.7%)	11 (5.3%)	0.606

Data are presented as number (%). Values in bold indicate *P* < 0.05

*CD* Clavien**–**Dindo classification system, *ICU* intensive-care unit, *LOS* length of stay

**Table 3:** Effects of preoperative hyponatremia in predicting the postoperative outcomes in matched cohort

Outcomes	Logistic regression analyses		
	OR	95% CI	*P* value
Primary outcome			
CD IV and V complications	2.082	1.041–4.164	**0.038**
Secondary outcomes			
CD II or greater complications			
Cardiovascular	1.180	0.595–2.339	0.636
Respiratory	1.740	0.874–3.463	0.115
Neurological	1.700	0.507–5.707	0.390
Renal	1.117	0.365–3.421	0.847
Hepatic	0.495	0.055–4.487	0.532
Thromboembolic	0.490	0.102–2.351	0.373
Infectious	2.158	1.138–4.091	**0.018**
Gastrointestinal	0.923	0.466–1.828	0.818
ICU admission after surgery	1.042	0.642–1.691	0.869
Prolonged LOS in hospital	0.929	0.545–1.584	0.787
Adverse discharge disposition	1.292	0.486–3.438	0.607

Values in bold indicate *P* < 0.05

*OR* odds ratio, *CI* confidence interval, *CD* Clavien**–**Dindo classification system, *ICU* intensive-care unit, *LOS* length of stay

### Secondary outcomes after matching

Compared with patients with normal serum [Na^+^] levels, hyponatremic patients had a higher incidence of infectious complications (21.2% vs. 11.1%, *P* = 0.017; Table 2). The logistic regression analysis suggested that preoperative hyponatremia was predictive of an increased risk of infectious complications (OR 2.158, 95% CI 1.138–4.091, *P* = 0.018; Table 3).

### Sensitivity analysis

We conducted a sensitivity analysis across the entire cohort (*n* = 1076) to verify the primary result. In the pre-matched cohort, there was a significantly higher incidence of CD IV and V complications in the hyponatremia group compared with the normal [Na^+^] group (20.5% vs. 7.1%, *P* < 0.001; Supplementary Table 4). After adjusting for the 11-item mFI score, ASA classification, CCI score, diabetes mellitus, arrhythmia, preoperative blood transfusion, hypoalbuminemia, type of surgery, emergency surgery, and intraoperative blood transfusion, preoperative hyponatremia remained an independent predictor for the development of life-threatening postoperative complication and mortality (adjusted OR 2.511, 95% CI 1.453–4.340, *P* = 0.001; Table 4 and Supplementary Table 5).

**Table 4:** Effect of preoperative hyponatremia in predicting CD IV and V complications in the entire cohort (sensitivity analysis)

	Univariate analysis		Multivariate analysis^a^	
	OR (95% CI)	*P* value	OR (95% CI)	*P* value
Preoperative hyponatremia	3.358 (2.029–5.559)	< 0.001	2.511 (1.453–4.340)	0.001

*OR* odds ratio, *CI* confidence interval

^a^After testing for collinearity, factors with *P* values < 0.05 in univariate analyses (including 11-item mFI score, ASA classification, CCI score, diabetes mellitus, arrhythmia, preoperative blood transfusion, hypoalbuminemia, type of surgery, emergency surgery, and intraoperative blood transfusion) were included in the multivariate logistic regression model to identify an association between preoperative hyponatremia and the primary outcome in the entire cohort. The multivariate logistic regression analysis was performed with the backward stepwise method

## Discussion

In this retrospective cohort study, we found that preoperative hyponatremia was associated with an increased risk of life-threatening complications and mortality in older patients undergoing digestive tract surgery. Furthermore, we determined that preoperative hyponatremia was associated with a higher risk of postoperative infectious complications.

In the present study, the prevalence of preoperative hyponatremia in the entire cohort was 11.3%; this was higher than the previously reported prevalence of preoperative hyponatremia in patients undergoing general surgical procedures (7.5%) [21]. This difference could be attributed to the fact that the participants included in the present study were all older patients who were ≥ 65 years of age. The prevalence of hyponatremia in older inpatients was reported to be 1.43-fold higher than that in the younger inpatient population [22]. Older people are predisposed to hyponatremia. The reasons for the age-related increased susceptibility to hyponatremia are multifactorial. The most prevailing cause of hyponatremia in older inpatients is SIADH [8]. SIADH is a disorder characterized by inappropriate secretion of antidiuretic hormone (ADH) in the absence of adequate stimuli, leading to impaired water excretion and hypotonic and euvolemic hyponatremia [2, 23]. In addition to the frequent causes (e.g., cancers, pulmonary or central nervous system diseases, and drugs), a cause of SIADH worth a special mention is the idiopathic factor, which is considered to be related to aging [9]. Other age-related changes in water-excretory capacity also contribute to hyponatremia. For example, the glomerular filtration rate [GFR] decreases with age, thereby potentially impairing the free water excretion. The total body water content declines in older people, resulting in a more significant fluctuation in [Na^+^] levels and an increased vulnerability to sodium disturbance. In addition, there is a reduced renal synthesis of prostaglandins in older individuals, which may potentiate the effects of AVP and further impair the ability to excrete a water load [24, 25]. Frailty, as a common geriatric syndrome characterized by reduced physiological reserves and loss of capacity to maintain homeostasis, may involve decreasing the tolerance to stresses on water homeostasis and impairing the compensatory mechanisms in case of sodium abnormality; therefore, it is not surprising that the frailty syndrome is associated with the development of hyponatremia [7]. In addition to hyponatremia, older people are also susceptible to hypernatremia due to age-related physiological changes, such as deficiency in thirst sensation and impairment of urine concentrating abilities.

In our target population, i.e., the geriatric patients scheduled for digestive tract surgery, two additional predisposing factors for hyponatremia might be the increased nonosmotic AVP secretion (stimulated by the volume depletion) and the reduced oral sodium intake [2, 3]. The volume depletion is due to gastrointestinal fluid losses (caused by vomiting or diarrhea) or the third spacing of fluids (caused by small bowel obstruction, hypoalbuminemia, etc.). The reduced oral sodium intake may be associated with reduced diet. Thus, it can be seen that the presence of preoperative hyponatremia might indicate more severe gastrointestinal symptoms or worse baseline status in this patient population. In accordance with the previous study [21], we found that patients with preoperative hyponatremia were typically older, had a higher ASA classification, had more comorbidities, had a higher rate of impaired ADLs, and were more likely to undergo emergency surgery. To minimize bias, we used the PSM method to balance these covariates and provide a more valid comparison between the two groups.

In the current study, the incidence of CD IV and V complications in the entire cohort was 8.6%. We adopted the Clavien–Dindo classification system, which distinguishes the severity of complications according to different levels of intervention, to evaluate the postoperative complications. This classification system is known to standardize the evaluation of surgical complications in an objective manner and improve comparability between different studies [19]. Previous studies reported that the incidence of CD IV and V complications after various digestive system surgeries ranged from 8.0 to 10.1% [26, 27]. The incidence of CD IV and V complications in our cohort was within the range reported by previous studies.

The relationship between preoperative hyponatremia and postoperative complications has not been validated in the older patient undergoing digestive tract surgery. Previous studies have confirmed the association between preoperative hyponatremia and mortality in heterogeneous cohorts of surgical patients. In a cohort study of 964,263 adult patients undergoing major surgery, Leung et al. reported that the incidence of 30-day perioperative mortality increased by 44% in hyponatremic patients when compared with patients with normal [Na^+^] levels [21]. In another study of 4370 patients receiving cardiac surgery, Crestanello et al. revealed that hyponatremia was independently associated with an increased hazard ratio for mortality [13]. Similar findings were also identified in pediatric surgical patients [14]. Regarding the impacts of hyponatremia on postoperative complications, however, there is some controversy [13, 16, 21]. Leung et al. found that preoperative hyponatremia was independently associated with a higher risk of perioperative major coronary events, wound infections, or pneumonia in adult patients undergoing major surgery [21]. Crestanello et al. reported that the hyponatremic patients had a higher incidence of postoperative renal failure requiring dialysis or pulmonary complications after cardiac surgery [13]. In contrast, Abola et al. found that hyponatremia was not associated with an increased risk of major morbidity in the patients following total knee arthroplasty [16]. These conflicting results might be attributed to the different study populations and a lack of standardization in complication evaluation. Furthermore, in reviewing the previous studies, we found that no research had examined the influences of preoperative hyponatremia on the postoperative outcomes in older patients undergoing digestive tract surgery. To address these knowledge gaps, we adopted two strategies. First, we focused exclusively upon a cohort of older patients (≥ 65 years of age) undergoing digestive tract surgery to investigate the relationship between hyponatremia and postoperative outcomes. Second, we adopted the standardized Clavien–Dindo classification system to evaluate postoperative complications; by this means, we could identify most complications and avoid down-rating of major morbidity.

Our results showed that preoperative hyponatremia was independently associated with an increased risk of life-threatening complications and mortality in older patients undergoing digestive tract surgery. There is still controversy as to whether links between hyponatremia and poor outcomes are causal or epiphenomenal. Based on our findings together with others [12–16, 21], preoperative hyponatremia might have direct effects on postoperative outcomes. The potential pathophysiologic mechanisms responsible for these effects might involve hyponatremia-related hypo-osmolality, low extracellular [Na^+^] concentration itself, and activation of the neurohormonal axis (including AVP, the renin–angiotensin–aldosterone system, and the sympathetic nervous system) [2, 5, 13, 28–30]. Sodium and its accompanying anions are the major osmotically active plasma solutes; the decrease of serum [Na^+^] concentration can directly cause a decrease in plasma tonicity and evoke the ECF shift into the cells, thus altering cell volume and threatening cell viability [2, 13]. This hyponatremia-related cellular dysfunction can disrupt the normal physiological functions of multiple organ systems, especially the central nervous system. The low extracellular [Na^+^] concentration, rather than hypo-osmolality, has been proved to inhibit ascorbic acid uptake into cells, increase the accumulation of reactive oxygen species, and induce oxidative stress responses. This pathophysiologic mechanism may accelerate the aging process of multiple organ systems and increase intolerance to stressors [28, 29]. Loss of intravascular volume (in hypovolemic hyponatremia) and loss of effective intravascular volume (in hypervolemic hyponatremia) can activate the neurohumoral axis, resulting in increased secretion of AVP, renin, angiotensin II, aldosterone, and catecholamines [2, 5]. Elevated levels of AVP and other above hormones might cause peripheral and renal vasoconstriction or enhance myocardial remodeling and fibrosis, thereby worsening cardiac function [5, 30]. However, the specific roles of these mechanisms in the adverse effects of hyponatremia need further studies to clarify.

In the present study, we also found that preoperative hyponatremia was significantly correlated with a higher risk of infectious complications. The impact of preoperative hyponatremia on the development of postoperative infectious complications remains controversial [13, 21, 31]. The conflicting results might be attributed to differences in the target population and the scope of infectious complications. In this study, we included all identified postoperative infections (classified into CD grade II or greater) in the analysis, including abdominal abscess, superficial or deep incisional infection, pulmonary infection, upper respiratory tract infection, urinary tract infection, sepsis, and infectious diarrhea. As far as we know, this is the first time to determine that preoperative hyponatremia was associated with an increased overall incidence of infectious complications in the perioperative settings. This finding warrants further attention and needs to be validated in other surgical populations.

Although the causal relationship between hyponatremia and adverse outcomes remains to be clarified, our results have clinical significance and highlight the need for further investigation. Our exploration reinforces the findings that preoperative hyponatremia can be regarded as a valid marker to predict adverse outcomes in perioperative settings. In addition, the current results, together with the evidence from in vitro and animal experiments [28, 29, 32–34], highlight the necessity of further studies to elucidate the causal relationship between hyponatremia and adverse outcomes in clinical settings. Furthermore, whether the tailored etiology-specific treatment of hyponatremia (such as fluid restriction, use of AVP receptor antagonists, or volume expansion with isotonic saline) can improve perioperative outcomes also deserves further explorations.

Our study had several limitations. First, due to the retrospective design, we did not have data on the geriatric assessment (such as cognitive ability, functional ability to perform advanced ADLs, etc.), factors related to the etiology of hyponatremia (such as plasma osmolality, glucose or lipid levels, medication history, etc.), the intraoperative or postoperative [Na^+^] concentration, and whether hyponatremia was corrected. However, these factors might confound the association between hyponatremia and outcomes. Although we used the PSM method to adjust for confounding factors, residual confounding remains inevitable. Second, given that the post-discharge outcomes were unavailable, the endpoints were limited to in-hospital adverse events, which might underestimate the total incidence of postoperative complications. Finally, this was a single-center study with a relatively limited sample size, which was further reduced after PSM. Although we performed a sensitivity analysis to verify the main results, multi-center studies with a larger sample size are needed to yield more definitive conclusions. Despite these, our results have clinical significance and generate hypotheses for further studies.

In conclusion, our results demonstrated that preoperative hyponatremia could predict an increased risk of life-threatening postoperative complications and mortality in older patients undergoing digestive tract surgery. Preoperative hyponatremia was also associated with a higher risk of postoperative infectious complications. These findings may help geriatric consultation teams identify high-risk older surgical patients and cooperate with surgical clinicians to provide tailored perioperative care and management for these older inpatients.

## Supplementary Information

Below is the link to the electronic supplementary material.

Supplementary file1 (DOCX 46 KB)

## Data Availability

The data that support the findings of the current study are available from the corresponding author on reasonable request.

## Code availability

All statistical analyses were performed with SPSS version 22 software (SPSS, Inc, Chicago, IL) and the free software package “R” version 2.15.3 including “Matchit” and “ROC” plugins.
