# Supplementary material for: Preoperative hyponatremia predicts complications in older patients undergoing digestive tract surgery: a propensity score matching analysis
Source: Eur Geriatr Med. 2021 Sep 23;13(2):493–503. doi: 10.1007/s41999-021-00559-4 (PMC9018643; doi:10.1007/s41999-021-00559-4)
Supplement: Supplementary file 1 — Supplementary file1 (DOCX 46 KB) [file 41999_2021_559_MOESM1_ESM.docx]

**Supplementary Files**

**Title:** Preoperative hyponatremia predicts complications in older patients undergoing digestive tract surgery: A propensity score matching analysis

**Target journal:** European Geriatric Medicine

**Authors:** Chun-Qing Li ^1*^, Chen Zhang ^1^, Fan Yu ^1^, Hao Kong ^1^, Chun-Mei Deng ^1^

**Affiliations of authors:**

^1^ Department of Anesthesiology and Critical Care Medicine, Peking University First Hospital, Beijing, China

**^*^ Corresponding Author:** Chun-Qing Li MD

**Email:** lichuntsing @163. com

**Supplementary Table 1** Clavien**-**Dindo classification of postoperative complications [19]

| Grade | Definition |
| --- | --- |
| Grade I | Any deviation from the normal postoperative course without the need for special interventions; complications that are managed with routine medication (such as analgesics, antiemetics, antipyretics, diuretics, and electrolytes) or physiotherapy, and wound infections that are managed at the bedside are classified as Grade I. |
| Grade II | Requiring total parenteral nutrition, blood transfusions, or pharmacological treatment with drugs other than those allowed for Grade I complications. |
| Grade III | Requiring surgical, endoscopic, or radiological intervention. |
| Grade IV | Life-threatening complications requiring intermediate care/intensive care unit (ICU) management excluding transient ischemic attacks. |
| Grade V | Death of a patient. |

**Supplementary Table 2** Diagnostic criteria of postoperative complications

| Complications | Diagnostic basis |
| --- | --- |
| **Cardiovascular** |  |
| Circulatory insufficiency | Requirement of positive inotropic agents and or vasopressors for more than 24 hours after surgery. |
| Acute myocardium infarction | The concentration of cardiac troponin I exceed the diagnostic criteria for myocardial infarction, together with new Q waves (lasts for 0.03 s at least) or continuous (4 days) abnormal ST‐T segment. |
| Angina | Symptoms of myocardial ischemia, without elevated cardiac biomarkers, with or without ECG changes that indicate ischemia. |
| New-onset arrhythmia | Confirmed by 12‐lead electrocardiogram and necessitated medical treatment and/or cardioversion. |
| Acute heart failure or exacerbation of congestive heart failure  Cardiac arrest | New-onset orthopnea with evidence of fluid retention (i.e., elevated jugular venous pressure, evidence of pulmonary edema, and/or peripheral edema) and a plasma brain natriuretic peptide ≥400 pg/ml.  The absence of large artery pulsation and heart sound, subsequent loss of consciousness, respiratory arrest, dilated pupils, and even death, caused by the sudden stop of the heart beating. |
| **Respiratory** |  |
| Respiratory failure | PaO_2_ <60 mmHg on room air, a ratio of PaO_2_ to inspired oxygen fraction <300, or arterial oxyhemoglobin saturation measured with pulse oximetry <90% and requiring oxygen therapy. |
| Pleural effusion | Chest X-ray demonstrating blunting of the costophrenic angle, loss of the sharp silhouette of the ipsilateral hemidiaphragm in upright position, evidence of displacement of adjacent anatomical structures, or (in supine position) a hazy opacity in one hemithorax with preserved vascular shadows. |
| Atelectasis | Lung opacification with a shift of the mediastinum, hilum, or hemidiaphragm toward the affected area, and compensatory overinflation in the adjacent nonatelectatic lung. |
| Bronchospasm  Aspiration pneumonia | Confirmed by new-onset expiratory wheezing and necessitated treatment of bronchodilators.  Pneumonia caused by aspiration and reflux of gastric contents. |
| **Neurological** |  |
| Stroke | Persisted new focal neurologic deficit and confirmed by neurologic imaging. |
| Transient ischemic attack  Delirium | A sudden onset of focal neurologic signs and/or symptoms that lasted for less than 24 hours. No acute infarction was found in the cranial imaging examination.  Diagnosed by psychiatric symptoms of inattention and thinking disorder, or inattention and altered level of consciousness, with an acute onset or a fluctuation course. |
| **Renal** |  |
| Acute renal failure | New-onset renal failure that required renal replacement therapy. |
| Acute kidney injury | Increase in serum creatinine by ≥0.3 mg/dl (≥26.5 μmol/l) within 48 hours, or to ≥1.5 times baseline within seven days, or urine volume <0.5 ml/kg/h for at least 6 hours. |
|  |  |
| **Hepatic** |  |
| Acute hepatic injury | Total serum bilirubin ≥33 mol/L caused by new-onset hepatic dysfunction, requiring medical interventions such as liver-protective drugs. |
|  |  |
|  |  |
| **Thromboembolic** |  |
| Pulmonary embolism | Hypotension or shock suspected of pulmonary embolism, and meet one of the following: filling defect in any branch of the pulmonary artery in computed tomographic pulmonary angiogram or right ventricular overload in echocardiogram. For suspected pulmonary embolism without symptoms, a positive finding in computed tomographic pulmonary angiogram was required for diagnosis. |
| Venous thrombosis  Disseminated intravascular coagulation | Non-compressibility of one or more venous segments on B-mode ultrasonography.  Diagnosed by abnormal bleeding symptoms, and more than three anomalies in the following items: platelet <100×10^9^/L or progressive decline; fibrinogen <1.5 g/L or progressive decline or >4 g/L; plasma fibrin degradation product (FDP) >20 mg/L or D-dimer level increased or positive, or 3P test (plasma protamine paracoagulation test) positive; prothrombin time (PT) shorter or longer than 3 s or activated partial thromboplastin time (APTT) shorter or longer than 10 s. |
| **Infectious**  Sepsis  Abdominal abscess | Two or more criteria of systemic inflammatory response syndrome, with known infection and new-onset dysfunction of at least one organ/system.  Diagnosed according to the significantly increased white blood cell count, clinic presentations of high fever, abdominal pain/distension, and peritoneal/pelvic effusion confirmed by imaging examination; or the purulent fluid extracted by puncture and bacteria cultured from the fluid. |
| Superficial or deep incisional infection  Pulmonary infection  Upper respiratory tract infection  Urinary tract infection  Infectious diarrhea  **Gastrointestinal** | Confirmed by pus expressed from the superficial or deep incision and bacteria cultured from the pus.  Receiving antibiotics for suspected respiratory infection and meet at least one of the following criteria: new or changed sputum, new or changed lung opacities, fever, leukocyte count >12×10^9^/L.  Acute inflammation of the airway from the nasal cavity to the larynx, diagnosed by any two of the following items: runny nose, sore throat, sneeze, stuffy nose, cough, or fever; and necessitated antiviral therapy.  Confirmed by urinalysis and urine culture and necessitated antibiotic therapy.  Infectious diarrhea requiring antibiotics; intestinal fluid excretion ≥ 2000 ml/day accompanied by dehydration or electrolyte abnormality. |
| Anastomotic fistula | Extravasation of contrast agent in the body cavity or retroperitoneal space during imaging examination, or anastomosis-associated abdominal pain and peritonitis with changes in laboratory indicators, or perianastomotic abscess. |
| Pancreatic fistula  Ileus | Increase of amylase level in drainage fluid ≥3 times the upper limit of institutional criteria, on or after postoperative day 3.  Lack of bowel movement, flatulence, and requirement of intravenous fluid therapy for more than one week after surgery. |
| Anastomotic stenosis | Diagnosis supported by imaging examination or endoscopy, and excluded the presence of gastrointestinal dysfunction and mechanical obstruction. |
| Gastrointestinal function recovery disorders | Manifested by nausea, abdominal distension, the prolonged recovery time of exhaust and defecation, intolerance to oral feeding, a requirement of intravenous fluid therapy for more than one week after surgery, and ruled out stenosis and obstruction. |
| Gastrointestinal hemorrhage | Hematemesis, black stool, changes in drainage, changes in hemoglobin, and other direct or indirect evidence suggested the possibility of gastrointestinal bleeding. |
|  |  |
|  |  |

**Supplementary Table 3** Baseline and intraoperative characteristics of the entire cohort

| Covariates | All | Hyponatremia | Normal sodium | ASD ^a^ |
| --- | --- | --- | --- | --- |
|  | (n=1076) | (n=122) | (n=954) |  |
| **Preoperative factors** |  |  |  |  |
| Age, years | 73.9±6.3 | 76.5±6.6 | 73.6±6.2 | **0.444** |
| Female | 421 (39.1%) | 53 (43.4%) | 368 (38.6%) | 0.098 |
| Body mass index, kg/m^2^ | 23.4±3.4 | 22.1±3.5 | 23.6±3.4 | **0.419** |
| Modified frailty index |  |  |  |  |
| 0.00 | 214 (19.9%) | 17 (13.9%) | 197 (20.6%) | **0.193** |
| 0.09 | 342 (31.8%) | 37 (30.3%) | 305 (32.0%) | 0.036 |
| 0.18 | 259 (24.1%) | 30 (24.6%) | 229 (24.0%) | 0.014 |
| 0.27 | 162 (15.1%) | 20 (16.4%) | 142 (14.9%) | 0.041 |
| 0.36 | 67 (6.2%) | 14 (11.5%) | 53 (5.6%) | **0.185** |
| ≥0.45 | 32 (3.0%) | 4 (3.3%) | 28 (2.9%) | 0.019 |
| ASA classification |  |  |  |  |
| I/II | 609 (56.6%) | 51 (41.8%) | 558 (58.5%) | **0.339** |
| III | 421(39.1%) | 60 (49.2%) | 361 (37.8%) | **0.231** |
| IV/V | 46 (4.3%) | 11 (9.0%) | 35 (3.7%) | **0.219** |
| CCI score | 2 (2, 3) | 2 (0, 4) | 2 (2, 3) | 0.075 |
| NYHA classification |  |  |  |  |
| I | 491 (45.6%) | 38 (31.1%) | 453 (47.5%) | **0.341** |
| II | 532 (49.4%) | 73 (59.8%) | 459 (48.1%) | **0.236** |
| III | 53 (4.9%) | 11 (9.0%) | 42 (4.4%) | **0.185** |
| Impaired ADLs ^b^ | 312 (29.0%) | 53 (43.4%) | 259 (27.1%) | **0.346** |
| Recent weight loss ^c^ | 221 (20.5%) | 28 (23.0%) | 193 (20.2%) | 0.068 |
| Hypertension | 553 (51.4%) | 70 (57.4%) | 483 (50.6%) | **0.137** |
| Diabetes mellitus | 263 (24.4%) | 33 (27.0%) | 230 (24.1%) | 0.067 |
| Coronary artery disease | 205(19.1%) | 21 (17.2%) | 184 (19.3%) | 0.054 |
| Arrhythmia ^d^ | 92 (8.6%) | 15 (12.3%) | 77 (8.1%) | **0.139** |
| Previous stroke | 189 (17.6%) | 22 (18.0%) | 167 (17.5%) | 0.013 |
| Pulmonary diseases ^e^ | 108 (10.0%) | 15 (12.3%) | 93 (9.7%) | 0.083 |
| Hepatic insufficiency ^f^ | 59 (5.5%) | 18 (14.8%) | 41 (4.3%) | **0.363** |
| Renal dysfunction ^g^ | 46 (4.3%) | 8 (6.6%) | 38 (4.0%) | **0.116** |
| Preoperative infections ^h^ | 90 (8.4%) | 16 (13.1%) | 74 (7.8%) | **0.174** |
| Hypoalbuminemia ^i^ | 506 (47.0%) | 86 (70.5%) | 420 (44.0%) | **0.556** |
| Anemia ^j^ | 320 (29.7%) | 56 (45.9%) | 264 (27.7%) | **0.384** |
| Preoperative transfusion | 68 (6.3%) | 12 (9.8%) | 56 (5.9%) | **0.133** |
| **Intraoperative factors** |  |  |  |  |
| Indication for surgery |  |  |  |  |
| Malignancy | 777 (72.2%) | 72 (59 .0%) | 705 (73.9%) | **0.320** |
| Benign | 299 (27.8%) | 50 (41.0%) | 249 (26.1%) | **0.320** |
| Type of surgery |  |  |  |  |
| Simple general surgeries ^k^ | 81 (7.5%) | 7 (5.7%) | 74 (7.8%) | 0.084 |
| Gastric | 168 (15.6%) | 13 (10.7%) | 155 (16.2%) | **0.162** |
| Intestinal | 671 (62.4%) | 77 (63.1%) | 594 (62.3%) | 0.017 |
| Hepatopancreatobiliary | 156 (14.5%) | 25 (20.5%) | 131 (13.7%) | **0.181** |
| Emergency surgery | 151 (14.0%) | 34 (27.9%) | 117 (12.3%) | **0.397** |
| Surgical approach |  |  |  |  |
| Laparotomy | 290 (27.0%) | 40 (32.8%) | 250 (26.2%) | **0.145** |
| Laparoscopy | 786 (73.0%) | 82 (67.2%) | 704 (73.8%) | **0.145** |
| Anesthetic method |  |  |  |  |
| General aesthesia | 600 (55.8%) | 89 (73.0%) | 511 (53.6%) | **0.411** |
| Others ^l^ | 476 (44.2%) | 33 (27.0%) | 443 (46.4%) | **0.411** |
| Duration of surgery, min | 182 (133, 247) | 166 (123, 239) | 184 (134, 248) | 0.083 |
| Intraoperative transfusion | 74 (6.9%) | 16 (13.1%) | 58 (6.1%) | **0.239** |

Data are presented as number (%), mean ± SD, or median (interquartile range).

^a^ An ASD ≥0.1 was considered unbalanced [20]. ASDs in bold indicate those of ≥0.1.

^b^ Requiring partial or total assistance from other people for basic ADLs (such as feeding, bathing, grooming, continence, transfers, etc.); evaluated with the Barthel Index scale [18].

^c^ Unintentional body weight loss ≥10% of the baseline weight within 6 months, or ≥5% within 3 months, or ≥2% within 1 month.

^d^ Arrhythmia that required medical or interventional therapy.

^e^ Include chronic obstructive pulmonary disease and asthma.

^f^ Defined as Child-Pugh class B and C.

^g^ Defined as eGFR <45 ml/min/1.73 m² or on dialysis.

##### ^h^ Include preoperative intra-abdominal infection and respiratory infection.

^i^ Defined as preoperative serum albumin level <40 g/L.

^j^ Diagnosed according to the last laboratory test results before surgery: Male: <120 g/L, female <110 g/L.

^k^ Defined as low-risk, less-damaging, and 23-hour-stay digestive tract surgeries, such as laparoscopic cholecystectomy, hepatic cyst fenestration, hernia repair, and appendectomy.

^l^ Include combined epidural-general anesthesia, combined peripheral nerve block-general anesthesia, and epidural/combined spinal-epidural anesthesia.

ASD, absolute standardized difference; ASA, American Society of Anesthesiologists; CCI, Charlson Comorbidity Index; NYHA, New York Heart Association; ADLs, activities of daily living.

**Supplementary Table 4**  Postoperative outcomes of the entire cohort

| Outcomes | All | Hyponatremia | Normal sodium | *P* value |
| --- | --- | --- | --- | --- |
|  | (n=1076) | (n=122) | (n=954) |  |
| **Primary outcome** |  |  |  |  |
| CD IV and V complications | 93 (8.6%) | 25 (20.5%) | 68 (7.1%) | **<0.001** |
| CD IV complications | 76 (7.1%) | 18 (14.8%) | 58 (6.1%) | **<0.001** |
| CD V complication | 17 (1.6%) | 7 (5.7%) | 10 (1.0%) | **<0.001** |
| **Secondary outcomes** |  |  |  |  |
| CD II or greater complications |  |  |  |  |
| Cardiovascular | 96 (8.9%) | 21 (17.2%) | 75 (7.9%) | **0.001** |
| Respiratory | 89 (8.3%) | 23 (18.9%) | 66 (6.9%) | **<0.001** |
| Neurological | 32 (3.0%) | 7 (5.7%) | 25 (2.6%) | 0.104 |
| Renal | 26 (2.4%) | 8 (6.6%) | 18 (1.9%) | **0.004** |
| Hepatic | 20 (1.9%) | 3 (2.5%) | 17 (1.8%) | 0.869 |
| Thromboembolic | 35 (3.3%) | 2 (1.6%) | 33 (3.5%) | 0.426 |
| Infectious | 127 (11.8%) | 27 (22.1%) | 100 (10.5%) | **<0.001** |
| Gastrointestinal | 142 (13.2%) | 19 (15.6%) | 123 (12.9%) | 0.410 |
| ICU admission after surgery | 302 (28.1%) | 52 (42.6%) | 250 (26.2%) | **<0.001** |
| Prolonged LOS in hospital | 286 (26.6%) | 33 (27.0%)) | 253 (26.5%) | 0.901 |
| Adverse discharge disposition | 45 (4.2%) | 9 (7.4%) | 36 (3.8%) | 0.061 |

Data are presented as number (%). Values in bold indicate *P*< 0.05.

CD, Clavien**-**Dindo classification system; ICU, intensive care unit; LOS, length of stay.

**Supplementary Table 5** Predictors of Clavien**-**Dindo IV and V complications in the entire cohort

|  | Univariate analyses | |  | Multivariate analysis ^a^ | |
| --- | --- | --- | --- | --- | --- |
| Variables | OR (95% CI) | *P* Value |  | OR (95% CI) | *P* Value |
| Preoperative hyponatremia | 3.358 (2.029-5.559) | <0.001 |  | 2.511 (1.453-4.340) | 0.001 |
| Modified frailty index |  |  |  |  |  |
| 0.00 | Reference |  |  |  |  |
| 0.09 | 2.031 (0.939-4.393) | 0.072 |  | --- | --- |
| 0.18 | 1.906 (0.849-4.278) | 0.118 |  | --- | --- |
| 0.27 | 2.496 (1.074-5.804) | 0.034 |  | --- | --- |
| 0.36 | 4.474 (1.767-11.331) | 0.002 |  | --- | --- |
| ≥0.45 | 8.913 (3.216-24.706) | <0.001 |  | --- | --- |
| ASA classification |  |  |  |  |  |
| I/II | Reference |  |  | Reference |  |
| III | 2.839 (1.744-4.623) | <0.001 |  | 2.229 (1.338-3.712) | 0.002 |
| IV/V | 12.636 (6.198-25.760) | <0.001 |  | 6.895 (3.071-15.483) | <0.001 |
| CCI score | 1.135 (1.035-1.245) | 0.007 |  | 1.135 (1.025-1.257) | 0.015 |
| Diabetes mellitus | 1.619 (1.026-2.553) | 0.038 |  | --- | --- |
| Arrhythmia ^b^ | 3.201 (1.833-5.590) | <0.001 |  | 2.213 (1.177-4.162) | 0.014 |
| Anemia ^c^ | 1.895 (1.229-2.922) | 0.004 |  | --- | --- |
| Preoperative transfusion | 2.179 (1.100-4.318) | 0.026 |  | --- | --- |
| Hypoalbuminemia ^d^ | 2.189 (1.406-3.409) | 0.001 |  | --- | --- |
| Type of surgery |  |  |  |  |  |
| Simple general and intestinal ^e^ | Reference |  |  | Reference |  |
| Gastric | 1.583 (0.901-2.779) | 0.110 |  | 2.024 (1.114-3.679) | 0.021 |
| Hepatopancreatobiliary | 2.165 (1.274-3.680) | 0.004 |  | 2.057 (1.151-3.676) | 0.015 |
| Duration of surgery, hour ^f^ | 1.206 (1.076-1.351) | 0.001 |  | --- | --- |
| Emergency surgery | 2.664 (1.631-4.349) | <0.001 |  | 2.061 (1.147-3.703) | 0.016 |
| Intraoperative transfusion | 3.012 (1.633-5.554) | <0.001 |  | --- | --- |

^a^ After testing for collinearity, factors with *P* values <0.05 in univariate analyses were included in the multivariate logistic regression model. The multivariate logistic regression analysis was performed with backward stepwise method.

^b^ Arrhythmia that required medical or interventional therapy.

^c^ Diagnosed according to the last laboratory test results before surgery: Male: <120 g/L, female <110 g/L. Anemia was not enrolled into the multivariate logistic regression model because it was correlated with hypoalbuminemia.

^d^ Defined as preoperative serum albumin level <40 g/L.

^e^ The incidences of Clavien**-**Dindo IV and V complications in the groups of simple general, gastric, intestinal, and hepatopancreatobiliary surgeries were 0% (0/81), 10.7% (18/168), 7.9% (53/671), and 14.1% (22/156), respectively. Therefore, the groups of simple general and intestinal surgeries were combined for analysis.

^f^ Not included in multivariate logistic regression model because of correlation with the type of surgery.

OR, odds ratio; CI, confidence interval; ASA, American Society of Anesthesiologists; CCI, Charlson Comorbidity Index.
